# Predicting online shopping addiction: a decision tree model analysis

**DOI:** 10.3389/fpsyg.2024.1462376

**Published:** 2025-01-08

**Authors:** Xueli Wan, Jie Zeng, Ling Zhang

**Affiliations:** College of Chemistry and Life Sciences, Chengdu Normal University, Chengdu, China

**Keywords:** online shopping addiction, c5.0 decision tree model, predictive analysis, behavioral addiction, academic procrastination, social anxiety, self-efficacy, psychological mechanisms

## Abstract

**Background:**

Online shopping addiction has been identified as a detrimental behavioral pattern, necessitating the development of effective mitigation strategies.

**Objective:**

This study aims to elucidate the psychological mechanisms underlying online shopping addiction through constructing and analyzing a C5.0 decision tree model, with the ultimate goal of facilitating more efficient intervention methods.

**Methodology:**

A comprehensive survey was conducted among 457 university students in Sichuan, China, utilizing validated psychometric instruments, including the Online shopping addiction Scale, College Academic Self-Efficacy Scale, College Students’ Sense of Life Meaning Scale, Negative Emotion Scale, Social Anxiety Scale, Sense of Place Scale, and Tuckman Procrastination Scale.

**Results:**

The predictive model demonstrated an accuracy of 79.45%, identifying six key factors predictive of online shopping addiction: academic procrastination (49.0%), sense of place (26.1%), social anxiety (10.1%), college students’ sense of life meaning (7.0%), negative emotions (7.0%), and college academic self-efficacy (0.9%).

**Conclusion:**

This pioneering study in online shopping addictiononline shopping addiction prediction offers valuable tools and research support for identifying and understanding this behavioral addiction, potentially informing future intervention strategies and research directions. This study provides research support for improving people’s understanding and management of behavioral addictions and promoting healthier online shopping habits.

## 1 Introduction

The ubiquity and efficiency of e-commerce platforms have been identified as pivotal factors contributing to the proliferation of online shopping behaviors. Empirical research by Salehi et al. (2012) and Childers et al. (2001) elucidated that the popularity of online shopping stems from its convenience, efficiency, and capacity to serve as a proxy for physical product inspection, thereby positioning it as a prevalent lifestyle choice. Corroborating these findings, Shanthi and Desti (2015) accentuated the convenience of online shopping, highlighting its facilitation of consumer decision-making processes and price comparison capabilities. In congruence with these perspectives, Noori (2019) and Kripesh et al. (2020) posited that the widespread adoption of online shopping is attributable to its convenience, efficiency, and the extensive availability of products and services across geographical boundaries. Moreover, Katta and Patro (2016), as well as Duarte et al. (2018), underscored accessibility and convenience as critical drivers in the paradigm shift toward online purchasing behaviors, noting their particular relevance for consumers navigating time constraints and demanding lifestyles. Collectively, these scholarly works substantiate the hypothesis that the convenience and efficiency inherent in online shopping modalities have significantly contributed to their widespread integration into contemporary consumer practices and lifestyles. This body of literature suggests a robust correlation between the perceived utility of e-commerce platforms and their increasing prevalence in consumer behavior patterns. Notwithstanding the apparent benefits of online shopping, excessive engagement in this behavior has been associated with many adverse consequences spanning physical, academic, and social domains. Empirical investigations have established a strong correlation between deteriorating physical health and excessive online shopping behaviors (Ko et al., 2020; Nawodya and Kumara, 2022; Niedermoser et al., 2021; Trotzke et al., 2015). Moreover, the proliferation of e-commerce transactions and the pursuit of convenience among students have been observed to exert substantial influence on their academic trajectories. This phenomenon potentially culminates in overindulgence in online shopping activities, thereby impeding academic progress and performance (Chelvarayan et al., 2021; Muilenburg and Berge, 2005; Wang Q. et al., 2022). Further exacerbating these concerns, Lee and Park (2008) and Triningtyas and Margawati (2019) have elucidated the intricate relationship between social conformity psychology and excessive online shopping behaviors. Their research underscores how conformity psychology can precipitate impulsive consumer behaviors and highlights the deleterious consequences of unregulated online shopping practices. These patterns may manifest as impulsive and compulsive shopping behaviors, potentially leading to the development of behavioral addictions. In summation, a growing body of literature suggests that excessive online shopping can precipitate a cascade of negative outcomes, including but not limited to physical health deterioration, academic regression, and the onset of behavioral addictions (Cojocariu et al., 2021; Nawodya and Kumara, 2022; Ti et al., 2022). These findings underscore the critical need for further research into the mechanisms underlying excessive online shopping behaviors and the development of targeted interventions to mitigate their adverse effects.

The pervasive nature of online shopping and its potential for overuse have garnered significant scholarly attention globally, precipitating a diverse nomenclature in the extant literature. Contemporary research employs various terminologies to describe this phenomenon, including “compulsive online buying,” “online shopping addiction,” “excessive online consumption,” and “excessive online shopping” (Adamczyk, 2021; Dittmar et al., 2007; Duroy et al., 2014; Jain et al., 2018; Lee et al., 2012). For this study, the term “online shopping addiction” has been adopted, predicated on several key considerations. Firstly, “online shopping” explicitly underscores the digital context of the behavior, a crucial factor in its addictive potential. Conversely, the term “consumption” lacks this specificity and fails to convey the addictive nature of the behavior. Furthermore, the use of “addiction” implies a more severe and clinically significant level of impairment compared to the relatively milder connotation of “excessive,” thereby providing a more precise and apt descriptor for the phenomenon under investigation. This terminological choice aligns with the study’s objective to examine the more severe end of the spectrum of online shopping behaviors and their associated psychological mechanisms.

Extant literature has identified several key factors influencing online shopping addiction, including academic procrastination, sense of place, and college academic self-efficacy (Geng et al., 2018). Additionally, social anxiety, college students’ sense of life meaning, and negative emotions have been recognized as significant contributors to this phenomenon (Li et al., 2022; Müller et al., 2019; Rose and Dhandayudham, 2014). Despite these findings, there remains a paucity of research exploring the combined effects of academic factors, sense of place, and negative emotions on online shopping addiction. From a methodological perspective, decision tree models, as sophisticated data mining algorithms within machine learning, offer high predictive accuracy and the ability to decompose complex decision-making processes into more interpretable components (Batra and Agrawal, 2018). However, the application of machine learning techniques, particularly decision tree models, in predicting online shopping addiction remains underexplored. This gap in the literature underscores the need for more comprehensive studies that integrate multiple predictive factors and leverage advanced analytical techniques to elucidate the complex interplay of variables contributing to online shopping addiction among college students. Such research has the potential to enhance our understanding of this behavioral phenomenon and inform the development of more effective intervention strategies.

This study adopts an objective-based approach with three specific research objectives, grounded in existing literature: (1) to develop and validate a decision tree model for predicting online shopping addiction among college students; (2) to identify and rank the relative importance of predictive factors; and (3) to examine the interactions among these factors in contributing to online shopping addiction.

The primary objective of this study is to investigate the efficacy of decision tree models in predicting online shopping addiction and to identify salient variables that serve as robust predictors of this behavioral phenomenon. By elucidating these predictive factors, this research aims to provide empirically-grounded guidance for developing and implementing targeted intervention and prevention strategies. The subsequent sections of this paper will present a comprehensive examination of the influencing factors, the conceptual framework of online shopping addiction, its underlying impact mechanisms, and the predictive models employed in this analysis. This multifaceted approach seeks to contribute to the existing body of knowledge by offering a nuanced understanding of online shopping addiction, thereby facilitating more effective and tailored approaches to mitigating its negative consequences among vulnerable populations. The integration of advanced statistical techniques with a thorough exploration of psychological and environmental factors promises to yield valuable insights into this increasingly prevalent form of behavioral addiction.

## 2 Literature review

This study’s theoretical foundation is built upon three established frameworks in behavioral addiction research. First, the Conceptual Model of Over-shopping (OSA) proposed by Rose and Dhandayudham (2014) provides the foundational understanding of online shopping addiction through key components including self-esteem, self-regulation, affective states, and cognitive factors. Second, the Stress and Coping Model (SCM) by Cassidy and Adair (2021) establishes the theoretical link between psychological distress factors (loneliness, rejection sensitivity) and shopping addiction. Third, the study draws on the theoretical principles of the Machine Learning Model (MLP) framework (Nawodya and Kumara, 2022), which demonstrates how consumer motivations interact with environmental factors in developing addictive behaviors. These complementary theoretical perspectives provide a comprehensive foundation for examining the multiple pathways and predictors of online shopping addiction.

### 2.1 Online shopping addiction

Historically, the conceptualization of addiction and impulse control disorders was narrowly circumscribed, primarily encompassing substance-related addictions such as drug or alcohol dependence. However, contemporary research has expanded this paradigm to include non-substance addictions (Griffiths, 2000; Kwon et al., 2013). Within this broader framework, “behavioral addiction” has emerged as a distinct category of non-substance addiction, characterized by impulse control disorders centered on specific behaviors, such as excessive exercise or compulsive shopping (Grant et al., 2010). The “online shopping addiction” concept was first introduced in 1998, rapidly evolving into a novel research domain (Young, 2017). While there is a consensus among scholars that online shopping addiction refers to an uncontrollable urge to engage in online purchasing behaviors, influenced by the frequency of quotidian online shopping activities and digital experiences (Duong and Liaw, 2022), some researchers conceptualize it as a compulsive manifestation of otherwise routine activities (Jain et al., 2018). The etiology of online shopping addiction is multifaceted, potentially influenced by factors such as low self-esteem, poor self-regulation, negative affective states, hedonistic tendencies, gender, social anonymity, and cognitive overload (Rose and Dhandayudham, 2014). Moreover, internet addiction and increased accessibility to online shopping platforms may exacerbate impulsive and compulsive buying behaviors, thereby impacting self-control mechanisms and shopping patterns (Jain et al., 2018). For this study, online shopping addiction is operationally defined as compulsive, excessive, and uncontrollable online purchasing behavior that engenders negative consequences, mediated by factors including poor self-regulation, emotional distress, and digital experiences.

Recent research has highlighted the evolving nature of online shopping addiction within the broader context of behavioral addictions. Prodanova and Chopdar (2024) demonstrate how the acceleration of e-commerce has created new pathways to addictive behaviors, particularly examining how app characteristics and smartphone addiction interplay in mobile shopping behavior. Supporting this technological perspective, Duong and Liaw (2022) found that daily online shopping duration and frequency significantly predict addiction tendencies, emphasizing how the accessibility of digital retail platforms has transformed traditional shopping addiction patterns. This emergence of novel addiction vectors necessitates updated theoretical frameworks that account for the rapidly changing digital retail landscape.

### 2.2 Measurement and models of online shopping addiction

Contemporary assessment of online shopping addiction predominantly relies on psychometric scale models. Researchers have developed specialized instruments to quantify online shopping addiction behaviors, such as the Compulsive Online Shopping Scale (COSS) introduced by Manchiraju et al. (2017). It is noteworthy, however, that Griffiths et al. (2016) elucidate that the COSS is not an entirely novel instrument but rather an adaptation of the original 28-item Bergen Shopping Addiction Scale (BSAS) developed by Andreassen et al. (2015). For this study, we employ the Online Shopping Addiction Scale (OSAS), designed by Zhao et al. (2017) and grounded in a generalized addiction model. The OSAS has demonstrated robust psychometric properties, with multiple studies confirming its sufficient internal consistency (Duong and Liaw, 2022; Gong et al., 2021; Tian et al., 2018). The selection of the OSAS as the primary measurement tool is based on its empirically validated reliability and its comprehensive approach to assessing the multifaceted nature of online shopping addiction. This enhances the validity and generalizability of our findings within the broader context of addiction research.

Extant literature has comprehensively examined the phenomenon of online shopping addiction, employing diverse methodological approaches to elucidate its manifestation in quotidian behavior. Researchers have utilized observational techniques and in-depth interviews to investigate this behavioral addiction. For instance, Paik et al. (2014) conducted a case study that revealed the potential for online addiction syndrome to precipitate transient psychotic symptoms during withdrawal periods, with antipsychotic medication demonstrating efficacy in rapidly ameliorating these symptoms. Further case-based research by Jiang et al. (2017) underscored the critical role of high levels of self-control in mitigating online shopping addiction among college students and attenuating attentional bias towards shopping-related stimuli. Augsburger et al. (2020) contributed to this body of knowledge by observing that shopping disorders are intricately linked to in-store and online purchasing behaviors, while highlighting that variations in diagnostic methodologies may impact the identification of previously overlooked high-risk groups, such as males and older adults. These empirical investigations collectively underscore the complex, multifaceted nature of online shopping addiction and emphasize the need for nuanced, demographically sensitive approaches to its diagnosis and treatment.

Contemporary research on online shopping addiction is underpinned by various theoretical frameworks, including the Conceptual Model of Over-shopping (OSA), Stress and Coping Model (SCM), Machine Learning Model (MLP), and Mediation Models. The OSA Conceptual Model, as elucidated by Rose and Dhandayudham (2014), encompasses multifaceted components such as low self-esteem, poor self-regulation, negative affective states, hedonistic pursuits, gender predisposition, social anonymity, and cognitive overload. This model posits that current internet shopping experiences may precipitate problematic behaviors along a continuum, with OSA representing its extreme manifestation (Jiang et al., 2017). The Stress and Coping Model, as delineated by Cassidy and Adair (2021), demonstrates a robust positive correlation between feelings of loneliness, rejection sensitivity, and shopping addiction, suggesting that shopping addiction may be symptomatic of underlying emotional difficulties. The Machine Learning Model (MLP), with its impressive 90.90% accuracy rate, offers a novel approach to detecting online shopping addiction by analyzing consumer motivations within the context of attractive features and facilities provided by the online shopping environment (Nawodya and Kumara, 2022). Finally, the Mediation Model, as proposed by Wang Q. et al. (2022), elucidates the significant positive relationships among academic procrastination, online shopping addiction, and negative emotions, with online shopping addiction serving as a mediator between academic procrastination and negative affective states. This diverse array of theoretical frameworks underscores online shopping addiction’s complex, multidimensional nature and highlights the need for interdisciplinary approaches in its study and treatment.

### 2.3 Predictive analysis methods for online shopping addiction

Extant literature on the prediction of online shopping addiction encompasses various methodological approaches and analytical techniques. Günüç and Keskin (2016) employed a multi-faceted analytical strategy, incorporating descriptive analysis, *t*-tests, analysis of variance, and a two-step cluster analysis to classify hedonic shopping scores, complemented by content analysis for qualitative data interpretation. This comprehensive approach facilitated an in-depth examination of qualitative and quantitative data on online shopping behaviors, elucidating factors contributing to online shopping addiction and related conceptual constructs. Duong and Liaw (2022) utilized hierarchical analysis to demonstrate a significant negative correlation between online experience and online shopping addiction while also identifying daily online shopping duration and frequency as significant predictors of addiction scores. Furthermore, Li et al. (2022) implemented a mediation analysis to reveal that elevated stress levels and insufficient social support heighten the susceptibility to online shopping addiction among university students, with social support serving as a protective factor against this behavioral addiction. Notwithstanding these valuable contributions, there remains a notable paucity of research employing decision tree models for the prediction of online shopping addiction, highlighting a significant gap in the current literature and underscoring the potential for novel insights through the application of this advanced machine learning technique.

### 2.4 Predictive factors for online shopping addiction

**Figure 1:**
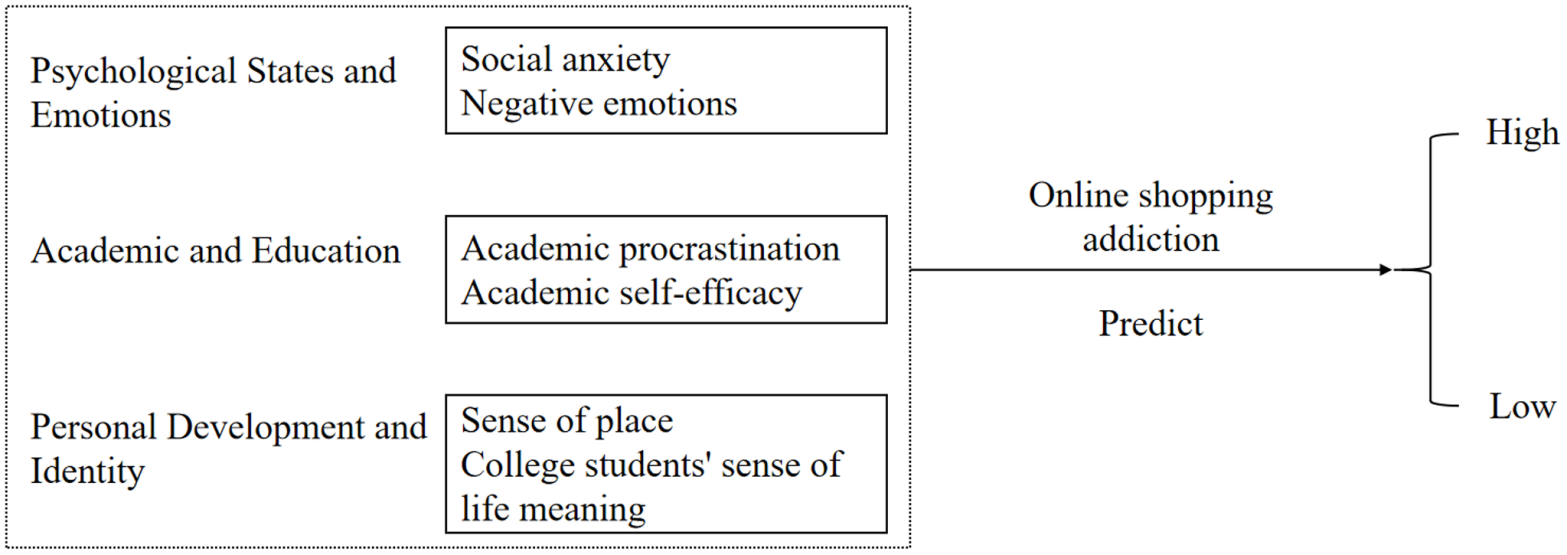
Hypothetical predictors of online shopping addiction.

In the extant literature, the application of decision tree models for predicting online shopping addiction remains relatively underexplored, particularly among university students. However, this approach offers several compelling advantages. Decision trees provide a transparent and easily interpretable structure, facilitating a nuanced analysis of addiction etiology. Their versatility in handling diverse data types, both numerical and categorical, renders them particularly suited to examining the multifaceted variables associated with online shopping behavior, such as personal income and shopping frequency (Kotsiantis, 2013; Sani et al., 2018; Loh, 2011). As a non-parametric method, decision trees circumvent assumptions about data distribution, making them adept at processing complex or irregular datasets (Ouyang et al., 2009). Moreover, these models excel in identifying salient variables influencing online shopping addiction, which is crucial for elucidating behavioral drivers and formulating preventative strategies. The adaptability of decision trees to new data and their robustness in managing missing data and outliers enhances their reliability in real-world applications (Khosravi et al., 2020). Given these attributes, the decision tree model emerges as a potent analytical tool for examining and forecasting online shopping addiction patterns among university students, underscoring the necessity of developing such a model based on established influential factors gleaned from the existing literature (see Figure 1).

This study posits College Academic Self-Efficacy, sense of place, and college students’ sense of life meaning as potential predictors of online shopping addiction. College Academic Self-Efficacy, in particular, has garnered substantial attention and empirical support as a critical factor in online shopping addiction. Saleh (2019) identified a positive correlation between College Academic Self-Efficacy and internet addiction among university students, establishing it as a key predictive variable for online shopping addiction. Corroborating this finding, Kuo and Belland (2019) demonstrated positive associations among computer self-efficacy, internet self-efficacy, and College Academic Self-Efficacy. Odacı (2013) further elucidated that College Academic Self-Efficacy in university students positively correlates with excessive internet use. Notably, research by Hassan et al. (2015), Keisidou et al. (2011), Milne et al. (2009), Ranganathan and Jha (2007), and Zamzuri et al. (2018) consistently emphasizes significant positive correlations between self-efficacy, internet self-efficacy, online shopping self-efficacy, and the propensity for online shopping behavior. These cumulative findings underscore the potential significance of College Academic Self-Efficacy in comprehending and addressing online shopping addiction among university students, thereby providing a robust theoretical foundation for developing targeted intervention strategies.

In examining the role of sense of place as a predictor of online shopping addiction behaviors, this study underscores its pivotal influence on consumer behavior, positing that it may even surpass the impact of consumers’ evaluations of shopping area characteristics. This finding accentuates the critical role of retail environments in fostering and stimulating a sense of place, thereby significantly shaping consumer behaviors (Van den Berg et al., 2021). Further empirical evidence suggests that consumers’ motivations, driven by the alluring features and facilities inherent in the online shopping experience, can serve as robust predictors of online shopping addiction (Nawodya and Kumara, 2022). These digital amenities may potentially enhance consumers’ sense of place within the virtual retail environment, consequently influencing their shopping behaviors and addiction propensities. Thus, a comprehensive understanding and strategic application of the sense of place concept emerge as crucial elements in the prevention and management of online shopping addiction, offering valuable insights for both researchers and practitioners in consumer psychology and digital retail management.

In examining the role of college students’ sense of life meaning as a predictor of online shopping addiction, extant research provides compelling insights. Zhang et al. (2015) demonstrate that for individuals with high impulsivity, both life meaning and self-esteem serve as adequate buffers against internet addiction. Otero-López et al. (2011) elucidate the mediating role of life satisfaction in the relationship between materialism and addictive buying behaviors, highlighting the importance of acquisition on female addictive buying tendencies. Further empirical analysis by Simmons (1980) reveals that the sense of life meaning is intricately linked to self-worth valuation, current and anticipated future satisfaction, and an emphasis on responsible self-control. These findings collectively underscore the complex interplay between life meaning, personal values, and addictive behaviors, offering a nuanced perspective on the potential protective factors against online shopping addiction among college students and providing valuable insights for developing targeted intervention strategies.

Empirical evidence suggests a nuanced relationship between personal values and online shopping behaviors among Chinese consumers. Wu et al. (2011) demonstrate that values oriented toward openness to change and self-enhancement positively influence online shopping behaviors, while conservation and self-transcendence values do not exert significant effects. Furthermore, Shahnaz and Karim (2014) elucidate the substantial impact of internet addiction on young individuals’ life satisfaction and engagement, with frequent users of social networking platforms exhibiting higher scores. Jiang et al. (2017) underscore the critical role of self-control in modulating attentional bias among individuals with online shopping addiction and high levels of attention concentration. These findings collectively emphasize the importance of integrating the sense of life meaning into prevention and intervention strategies for online shopping addiction among university students. Additionally, this study incorporates academic procrastination, social anxiety, and negative emotions as potential predictive factors for online shopping addiction, further expanding the multifaceted approach to understanding and addressing this phenomenon within the academic context.

Empirical investigations into academic procrastination as a predictive factor for online shopping addiction yield compelling results. Tras and Gökçen (2020) demonstrate a significant positive correlation between internet addiction in adolescents, academic procrastination, and social anxiety, with academic procrastination emerging as the most robust predictor. Specifically, a notable positive association between academic procrastination and online shopping addiction has been established. Moreover, Wang Q. et al. (2022) elucidate that online shopping addiction significantly predicts the generation of negative emotions. Further analysis by Nwosu et al. (2020) reveals internet addiction as the most salient predictor of academic procrastination among undergraduate students. Interestingly, while social media usage does not directly impact academic procrastination, it indirectly predicts its occurrence through the mediating effect of internet addiction. These findings collectively underscore the importance of considering academic procrastination as a key factor in comprehending and mitigating online shopping addiction among university students, offering valuable insights for developing targeted intervention strategies within the academic context.

Extant research suggests a significant relationship between social anxiety and online shopping addiction among university students. Li et al. (2022) demonstrates that students experiencing elevated stress levels and inadequate social support are substantially more susceptible to developing online shopping addiction. Conversely, social support emerges as a crucial protective factor, mitigating the onset of such addictive behaviors. Liu et al. (2022) further posit that implementing social sensing solutions may positively contribute to reducing shopping addiction. Concurrently, Madu (2020) elucidates that internet addiction is influenced by a complex interplay of neural plasticity, physiological, psychological, and social factors, underscoring the critical role of educational interventions in mitigating the deleterious effects of internet addiction on learning and social interaction. These findings collectively emphasize the imperative of addressing social anxiety and bolstering social support mechanisms in the prevention and management of online shopping addiction among university students, offering valuable insights for developing comprehensive intervention strategies within the academic milieu.

Current research on negative emotions as predictors of online shopping addiction reveals a complex interplay of psychological and social factors. Rose and Dhandayudham (2014) identify low self-esteem, poor self-regulation, negative emotional states, the pursuit of pleasure, female gender identity, social anonymity, and cognitive overload as correlates of online shopping addiction. Sun and Wu (2011) posit that emotional instability and lack of responsibility are predictive factors for internet addiction, which is closely associated with impulsive buying behaviors. Li et al. (2022) elucidates that academic distress, personal issues, and negative life events serve as triggers for Online Shopping Addiction Tendency (OSAT) among university students, while social support acts as a protective factor. Yao et al. (2013) demonstrate that psychological health symptoms and adjustment issues during the first year significantly predict internet addiction among Chinese male college students. Gupta et al. (2018) further establishes depression, anxiety, and stress as independent predictors of internet addiction in university populations. Brunelle and Grossman (2022) highlight the predictive role of high impulsivity, anxiety sensitivity, and lower levels of mindfulness in online compulsive buying behavior, suggesting the potential efficacy of mindfulness interventions. These findings collectively underscore the critical importance of addressing negative emotional factors in the prevention and management of online shopping addiction. Consequently, this study proposes several predictive factors for online shopping addiction among college students, including College Academic Self-Efficacy, sense of place, sense of life meaning, academic procrastination, social anxiety, and negative emotions, offering a comprehensive framework for understanding and addressing this multifaceted phenomenon.

## 3 Materials and methods

### 3.1 Participants

This investigation was conducted at a tertiary university in Sichuan Province, China. Prior to finalizing the research design, the investigators undertook exploratory focus interviews with a cohort of five volunteer participants to elucidate potential predictive factors of online shopping addiction. Prior to finalizing the research design, the investigators conducted exploratory focus interviews with five volunteer participants to inform the selection of potential predictive factors. These preliminary discussions identified three primary domains of interest: sense of life meaning among college students, negative emotional states, and academic procrastination tendencies. These qualitative insights helped refine our quantitative research framework and variable selection, enhancing the ecological validity and contextual relevance of the study within the specific sociocultural milieu of Chinese higher education.

This study employed a convenience sampling method to recruit participants, resulting in a total of 457 students completing the questionnaire. The sample comprised 229 s-year and 228 third-year students. Following data collection, researchers meticulously evaluated the validity of questionnaire responses, yielding 457 valid submissions. The gender distribution of participants reflected 114 males (24.9%) and 343 females (75.1%). This notable gender disparity is consistent with the predominantly female composition of teacher training colleges in China. The sample’s geographical representation included 329 rural students (72.0%) and 128 urban students (28.0%), a distribution that aligns with the prevalence of rural-origin students in Western Chinese universities. The age distribution of participants was as follows: 16 participants aged 18 or below (3.5%), 109 aged 19 (23.9%), 160 aged 20 (35.0%), 120 aged 21 (26.3%), 35 aged 22 (7.7%), and 17 aged 23 or above (3.7%). Research participants were recruited on a voluntary basis to complete anonymized questionnaires. This investigation was conducted in strict adherence to the ethical principles outlined in the Declaration of Helsinki and the American Psychological Association (APA) Code of Ethics, and the study received ethical approval from the Academic Ethics Committee of the School of Chemistry and Life Sciences at [Blinded] University. As a newly established undergraduate institution, the faculty-level committee was authorized for ethical review during the university-level committee’s formation period. All participants were university students aged 18–23. Informed consent was obtained from participants and their legal guardians. All data were anonymized to ensure confidentiality. Participation was voluntary, and withdrawal was allowed at any time without consequences. Special care was taken to protect minors, and interactions were conducted in a safe environment. Furthermore, all participants granted permission for the publication of the research findings. This demographic profile provides a comprehensive representation of the target population, facilitating a nuanced analysis of online shopping addiction predictors within the context of Chinese higher education.

### 3.2 Data collection and instruments

This research utilized a targeted design scheme and implemented data collection through an online questionnaire survey from September 2 to September 7, 2023. During class meetings, instructors presented students with a Quick Response (QR) code for the questionnaire as a learning task. The QR code, a machine-readable barcode containing substantial information, could be scanned using smartphones or tablets, redirecting students to the specific questionnaire link. In China, QR codes are ubiquitous, employed not only for accessing specific interfaces but also extensively utilized in financial transactions, identity verification, information retrieval, and various other applications. The QR code was linked to a webpage hosting the questionnaire for this study. To ensure the cross-cultural validity and accuracy of the questionnaire items, this study employed the back-translation method. The first researcher translated the scales from English to Chinese, followed by a second researcher who translated the text back to English. A third researcher then compared the original, translated, and back-translated versions to ensure both accuracy and consistency. Notably, prior to code distribution, class instructors comprehensively elucidated the study’s objectives and ensured voluntary participation from all students, a crucial step in maintaining research integrity and validity. This methodological approach underscores the study’s commitment to upholding ethical standards by prioritizing participants’ voluntary and informed consent throughout the data collection process, thereby enhancing the robustness and credibility of the research findings.

### 3.3 Materials

This study employed a comprehensive questionnaire comprising six sections with 94 items each, encompassing demographic information and multiple psychometric scales: the Online Shopping Addiction Scale, Tuckman Academic Procrastination Scale, Sense of Place Scale, Social Anxiety Scale, College Students’ Sense of Life Meaning Scale, Negative Emotion Scale, and College Academic Self-Efficacy Scale. Demographic data collected included gender, age, and urban or rural origin. Originally developed in English, these scales underwent a rigorous translation process for this study. The researchers implemented Brislin (1970) back-translation method to ensure translation quality and cross-cultural validity. This process involved the first researcher’s initial translation from English to Chinese, followed by a back-translation to English by a second researcher. Subsequently, a third researcher compared the original text, Chinese translation, and back-translated English version to assess translation accuracy. The translation underwent iterative refinement and optimization to ensure conceptual and linguistic equivalence across versions, thereby maintaining the psychometric integrity of the scales in the target language and cultural context.

#### 3.3.1 Online shopping addiction scale

This study utilized the Online Shopping Addiction Scale developed by Zhao et al. (2017), which comprises 18 items designed to assess compulsive online shopping behaviors. Exemplar items include “When I’m not shopping online, I’m always thinking about it,” “I often think about how to free up more time or money for online shopping,” and “Online shopping is significant to me.” The scale employs a 5-point Likert-type response format, ranging from 1 (strongly disagree) to 5 (strongly agree), yielding potential composite scores between 18 and 90. The scale demonstrated excellent internal consistency in the present investigation, with a Cronbach’s alpha coefficient of 0.959. This high reliability coefficient underscores the scale’s robust psychometric properties and its suitability for assessing online shopping addiction within the context of this study’s target population, thereby enhancing the validity and interpretability of the research findings.

#### 3.3.2 Tuckman academic procrastination scale

This study employed the Tuckman Academic Procrastination Scale (Tuckman, 1991) to assess participants’ propensity for academic procrastination. The scale comprises 16 items, including exemplars such as “Even when I know studying is important, I still procrastinate,” “I delay doing things I do not like to do,” and “When tasks have a deadline, I wait until the last minute before I complete them.” To maintain consistency with other measures in the study, the original 6-point response format was modified to a 5-point Likert-type scale, ranging from 1 (strongly disagree) to 5 (strongly agree). This adaptation resulted in potential composite scores ranging from 16 to 80. The scale demonstrated strong internal consistency in the current investigation, with a Cronbach’s alpha coefficient of 0.870. This robust reliability coefficient underscores the scale’s psychometric integrity and its appropriateness for assessing academic procrastination tendencies within the study’s target population, thereby enhancing the validity and interpretability of the research findings.

#### 3.3.3 Sense of place scale

This investigation utilized the Sense of Place Scale developed by Jorgensen and Stedman (2001) to assess participants’ place attachment and identity. The scale comprises 12 items, including exemplars such as “This place is related to me and reflects my existence,” “This place is closely related to me but cannot truly define my identity,” and “This place is closely related, and I can be my true self here.” Responses were recorded on a 5-point Likert-type scale, ranging from 1 (strongly disagree) to 5 (strongly agree), yielding potential composite scores between 12 and 60. The scale demonstrated acceptable internal consistency in the present study, with a Cronbach’s alpha coefficient of 0.716. While this reliability coefficient is lower than those of some other measures employed in the study, it still falls within the acceptable range for social science research. This psychometric property suggests that the scale provides a reasonably cohesive measure of sense of place within the context of this study’s target population, contributing valuable insights to the multifaceted exploration of factors influencing online shopping addiction among college students.

#### 3.3.4 Social anxiety scale

This study employed the Social Anxiety Subscale of the Self-Consciousness Scale (SASS-CS), initially developed by Fenigstein et al. (1975) and subsequently refined by Scheier and Carver (1985) to enhance clarity and accessibility. The scale comprises six items designed to assess social anxiety, including exemplars such as “In unfamiliar situations, I need a lot of time to overcome my shyness,” “When everyone is looking at me, I tend to mess things up,” and “I am easily embarrassed.” Responses were recorded on a 5-point Likert-type scale, ranging from 1 (strongly disagree) to 5 (strongly agree), yielding potential composite scores between 6 and 30. The scale demonstrated satisfactory internal consistency in the present investigation, with a Cronbach’s alpha coefficient of 0.775. This reliability coefficient indicates a robust level of inter-item consistency, supporting the scale’s utility in assessing social anxiety within the context of this study’s target population. The psychometric properties of the SASS-CS contribute to the overall validity and interpretability of the research findings, particularly in elucidating the relationship between social anxiety and online shopping addiction among college students.

#### 3.3.5 College students’ sense of life meaning scale

In the present study, we employed the Chinese version of the Meaning in Life Questionnaire (MLQ), as translated and adapted by Liu and Gan (2010), to assess participants’ sense of life meaning. This validated instrument comprises nine items (e.g., “I am searching for a purpose or mission in my life,” “My life lacks a clear purpose,” “I am seeking the meaning of my life”) and is structured along two dimensions: “Presence of Meaning in Life” and “Search for Meaning in Life.” To ensure methodological consistency, we modified the original 7-point Likert scale to a 5-point scale, ranging from 1 (strongly disagree) to 5 (strongly agree), yielding a potential score range of 9 to 45 for each participant. The scale’s psychometric properties were robust, with an internal consistency coefficient (Cronbach’s alpha) of 0.940 in the current study, indicating high reliability. This adaptation of the MLQ provides a culturally appropriate and psychometrically sound measure for assessing the construct of life meaning within our target population, facilitating a nuanced exploration of its relationship with online shopping addiction and other variables of interest.

#### 3.3.6 Negative emotion scale

In the current study, we utilized the Negative Emotion Scale, as revised by Antony et al. (1998), to assess participants’ negative emotional states. This psychometrically validated instrument comprises 21 items distributed across three distinct dimensions, encompassing various aspects of negative affect (e.g., “I do not seem to feel any pleasure or satisfaction at all,” “I feel my mouth is dry,” and “I find it difficult to calm down”). To ensure methodological consistency across our battery of measures, we adapted the original 4-point Likert scale to a 5-point scale, ranging from 1 (strongly disagree) to 5 (strongly agree). This modification resulted in a potential score range of 21–105 for each participant. The scale demonstrated excellent psychometric properties in our sample, with an internal consistency coefficient (Cronbach’s alpha) of 0.970, indicating high reliability. This robust measure of negative emotions provides a comprehensive assessment of affective states, facilitating a nuanced examination of their relationship with online shopping addiction and other variables of interest in our study.

#### 3.3.7 College academic self-efficacy scale

In the present study, we employed the College Academic Self-Efficacy Scale (CASES), developed by Owen and Froman (1988), to assess participants’ self-efficacy within academic contexts. This psychometrically validated instrument comprises nine items (e.g., “Compared to other students in my class, I want to have a better performance,” “I am confident that I can understand the teacher,” and “I want to have good performance in class”) designed to capture various dimensions of academic self-efficacy. Responses are recorded on a 5-point Likert scale, ranging from 1 (strongly disagree) to 5 (strongly agree), yielding a potential score range of 9–45 for each participant. The scale demonstrated robust psychometric properties in our sample, with an internal consistency coefficient (Cronbach’s alpha) of 0.905, indicating high reliability. This well-established measure of academic self-efficacy provides a comprehensive assessment of students’ beliefs in their academic capabilities, facilitating a nuanced examination of its relationship with online shopping addiction and other variables of interest in our study. The utilization of CASES enhances the validity and reliability of our findings within the context of academic performance and behavioral addictions among college students.

### 3.4 Design

This quantitative investigation aims to elucidate the underlying mechanisms of online shopping addiction. To achieve this objective, we utilized a questionnaire survey to collect data on students’ behaviors related to online shopping addiction and six predictive factors. We submitted the questionnaire materials and a comprehensive explanation of our survey design to the Ethics Review Committee of Chengdu Normal University and the principals of the participating schools. Separate meetings were convened to address research ethics and survey prerequisites. Upon receiving approval, we administered the questionnaire survey and collected responses from the student participants.

To process the survey data, we employed data mining methodologies, which possess the capability to reveal patterns of online shopping addiction. Within the humanities and social sciences domain, commonly utilized machine learning algorithms such as decision trees, k-nearest neighbors, neural networks, naive Bayes, and support vector machines have demonstrated efficacy in predicting students’ online shopping addiction tendencies. The decision tree algorithm was selected due to its transparent decision-making process, ability to handle non-linear relationships among psychological variables, and excellent tolerance for mixed data types. While other algorithms could be considered, decision trees’ interpretable nature makes them particularly valuable for counseling applications where understanding the reasoning process is essential.

Our selection of the decision tree method for this study was primarily predicated on the following considerations: (1) Decision tree models have been extensively applied in predicting students’ online shopping addiction propensities, and (2) they generate easily interpretable rules. Specifically, decision trees establish classification rules through sample training, producing a top-down chart that facilitates comprehension (Mining, 2006). In this hierarchical structure, the decision tree comprises a root node, several internal nodes, and multiple leaf nodes. The root node and internal nodes represent respective testing conditions (i.e., classification criteria), while the leaf nodes denote the final output. Rules can be inferred based on the tree structure formed by each node (Mitchell and Learning, 1997). (3) Furthermore, decision tree algorithms exhibit robust tolerance for multicollinearity and can effectively handle complex relationships among predictor variables. A classification decision tree is employed when predictor variables are categorical, whereas a regression decision tree is appropriate for continuous predictor variables (Miguéis et al., 2018). Given that our study aims to determine whether students exhibit high or low levels of online shopping addiction, we implemented the classification decision tree algorithm to construct the predictive model and analyze the influence of each factor in predicting online shopping addiction behaviors.

### 3.5 Construction of the decision tree

The optimal splitting variables and thresholds for the decision tree were determined based on the information entropy reduction rate criterion. Information entropy, a measure of the degree of impurity in a dataset, is defined as Equation 1 (Mitchell and Learning, 1997):


(1) $$\mathrm{Entropy}\left(D\right)=-\overset{m}{\underset{k=1}{\sum }}P_{k}\log_{2}P_{k}$$

Where D is the training dataset with a sample size of m, and $P_{k}$ is the probability of each class of samples. The information gain ratio is used to assess the difference in information entropy of the dataset under different classification methods. If the variable C is chosen to divide the dataset D into n subsets, then the information gain ratio is defined as Equation 2 (Quinlan, 1997):


(2) $$\mathrm{Gain}\;\mathrm{ratio}\;\left(D,C\right)=\frac{\mathrm{Entropy}\left(D\right)-\mathrm{Entropy}\left(D|C\right)}{\mathrm{Entropy}\left(C\right)}$$

The C5.0 algorithm selects attributes with the highest information gain ratio as splitting points and then creates multiple branches based on the values of that attribute, resulting in the generation of multiple subsets. This selection process is repeated until the last subset contains data of only the same category, thus achieving inductive classification of the data (Che et al., 2011). This methodological approach enables constructing a decision tree model that optimally classifies the dataset based on the most informative attributes, providing a robust framework for analyzing and predicting online shopping addiction behaviors among the study participants.

#### 3.5.1 Pruning of the decision tree

We implemented a post-pruning strategy by iteratively pruning the leaf nodes. Following the initial construction of the decision tree, we recursively traversed the dataset to each leaf node and computed the mean squared error for datasets both with and without the leaf nodes. The pruning decision was based on a comparative analysis of these error rates; if the mean squared error decreased after pruning, the node was eliminated, whereas it was retained if no improvement was observed (Quinlan, 1986). This pruning process serves to enhance the model’s generalizability and mitigate overfitting.

#### 3.5.2 Evaluation of the decision tree

We employed a data partitioning approach to assess our model’s robustness and predictive capability. We randomly selected 68% of the sample data (*n* = 311) as the training set, reserving the remaining 32% (*n* = 146) as the test set. The quality of the model was evaluated using three key metrics: accuracy, precision, and recall (Han et al., 2019). Accuracy is defined as the proportion of correctly classified samples relative to the total number of samples. Precision quantifies the proportion of accurate positive samples among the positive predictions in the results, providing insight into the model’s ability to avoid false positives. Recall, also known as sensitivity, gauges the proportion of actual positive samples accurately predicted by the model, indicating its effectiveness in identifying true positives. These metrics collectively provide a comprehensive assessment of the decision tree’s performance and predictive power in classifying online shopping addiction behaviors.

### 3.6 Data analysis and data encoding

We conducted comprehensive statistical analyses using SPSS 22.0 for descriptive statistics and IBM SPSS Modeler 18.0 for decision tree modeling. Initially, descriptive statistical analysis was employed to elucidate the frequency and principal characteristics of students’ online shopping addiction behaviors and associated predictor variables. Subsequently, we implemented decision tree analysis utilizing the C5.0 algorithm, representing a significant advancement over its predecessors, the ID3 and C4.5 algorithms introduced by Quinlan (1986) and Witten and Frank (2002). The C5.0 algorithm is renowned for its efficacy in handling large datasets, superior processing speed, and enhanced predictive accuracy (Xiong, 2011), marking it as a notable development in machine learning.

To facilitate the decision tree analysis, we categorized the sample into high and low online shopping addiction groups based on a 60% threshold. The predictor variables, including college student ethnic identity, negative emotions, and other nominal or continuous variables, were transformed into binary variables according to predetermined criteria (see Table 1). This binary encoding process optimizes the decision tree’s ability to discern meaningful patterns and relationships within the dataset, thereby enhancing the model’s predictive capabilities and interpretability. This methodological approach enables a nuanced examination of the factors contributing to online shopping addiction among college students, leveraging advanced statistical techniques to uncover complex relationships and predictive patterns within the data.

**Table 1:** Variable encoding and descriptive statistics.

**Variable**	**Encoding**	**Count**	**Percentage**
Online shopping addiction	0 = low	324	70.59%
	1 = high	133	29.10%
Tuckman academic procrastination	0 = low	344	75.27%
	1 = high	113	24.73%
Sense of place	0 = low	276	60.39%
	1 = high	181	39.61%
Social anxiety	0 = low	168	36.76%
	1 = high	289	63.24%
College students’ sense of life meaning	0 = low	69	15.10%
	1 = high	388	84.90%
Negative emotion	0 = low	381	83.37%
	1 = high	76	16.63%
College academic self-efficacy	0 = low	198	43.33%
	1 = high	259	56.67%

## 4 Results

### 4.1 Descriptive statistics

We conducted descriptive statistical analysis on this study’s continuous and ordinal variables, with the results presented in Table 2. Students exhibited a high level of online shopping addiction behavior, with a mean score of 3.11 (standard deviation = 0.923), exceeding 60% of the maximum score. This suggests that the majority of students experienced a high level of online shopping addiction. A threshold of 60% of the maximum score was utilized as the coding criterion for each variable.

**Table 2:** Descriptive statistics

Variable	Full score	Mean value	Standard deviation	60% of the full score
Tuckman academic procrastination	5	2.9884	0.6526	3
Sense of place	5	3.30999	0.4670	3
Social anxiety	5	3.6163	0.7302	3
College students’ sense of life meaning	5	4.2188	0.6765	3
Negative emotion	5	2.5638	0.9662	3
College academic self-efficacy	5	3.6676	0.6589	3
Online shopping addiction	5	3.1129	0.9226	3

The associations among variables were evaluated using Pearson (1897) product–moment correlation coefficient, with the results presented in Table 3. Tuckman academic procrastination, sense of place, social anxiety, college student life meaning, negative emotions, and College Academic Self-Efficacy demonstrated statistically significant positive correlations with online shopping addiction. These findings provide preliminary evidence for the interrelationships among the examined variables and their potential influence on online shopping addiction behaviors.

**Table 3:** Pearson’s *r* of the variables.

	**TAP**	**SP**	**SA**	**CSSLM**	**NE**	**CASE**	**OSA**
TAP	1						
SP	0.039	1					
SA	0.314	0.141	1				
CSSLM	−0.145	0.267	0.016	1			
NE	0.502	0.108	0.343	−0.082	1		
CASE	−0.056	0.393	−0.011	0.299	0.069	1	
OSA	0.488	0.308	0.464	0.221	0.426	0.196	1

TAP, Tuckman academic procrastination; SP, sense of place; SA, social anxiety; CSSLM, college students’ sense of life meaning; NE, negative emotion; CASE, college academic self-efficacy; OSA, online shopping addiction. *p < 0.05, **p < 0.01.

### 4.2 Predictive analysis of online shopping addiction

**Figure 2:**
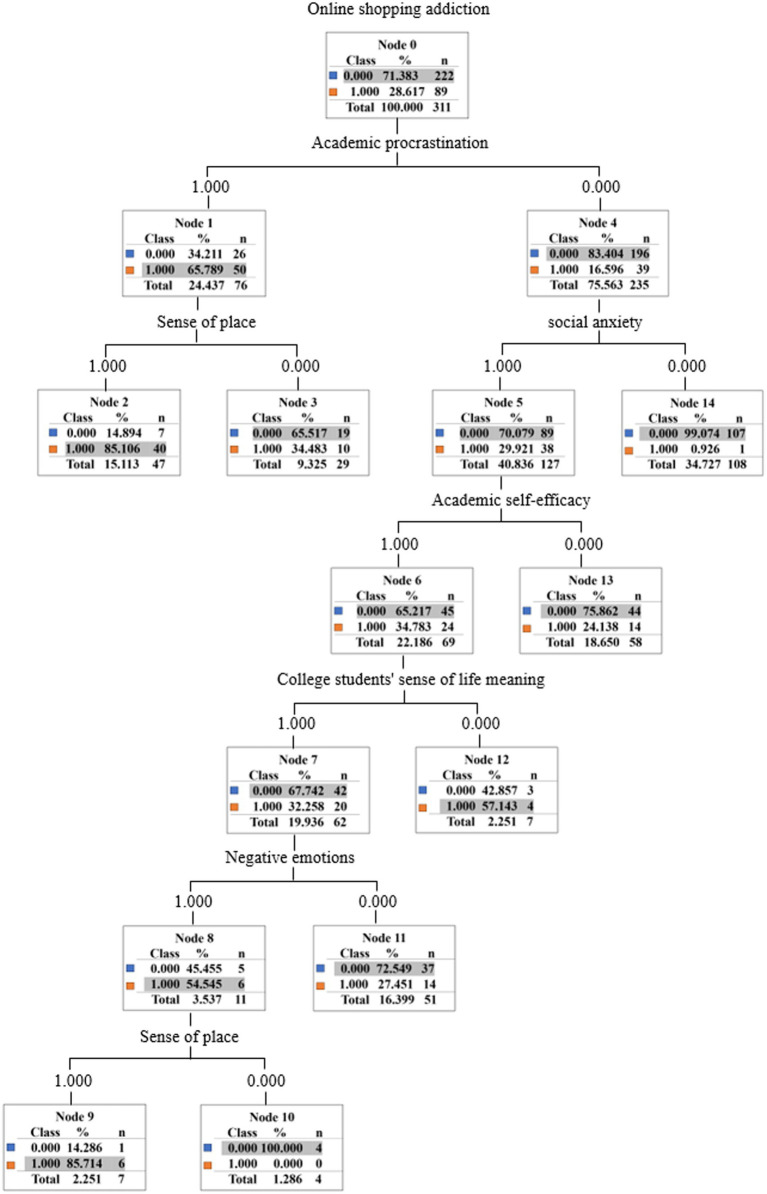
Predictive model of online shopping addiction.

The decision tree model for predicting online shopping addiction, illustrated in Figure 2, reveals a hierarchical structure of predictive factors. Academic procrastination emerges as the primary predictor, with students exhibiting high levels subsequently assessed based on their sense of place. This secondary predictor demonstrates high classification accuracy (85.11%) when elevated, whereas a low sense of place correlates with reduced online shopping addiction tendencies (34.48%). For students displaying low levels of academic procrastination, the model bifurcates into two branches predicated on social anxiety levels.

Social anxiety serves as the tertiary predictor, with high social anxiety necessitating further evaluation through four additional predictors. Among students with a low sense of life meaning, online shopping addiction prevalence is notably higher (57.14%). The model incorporates negative emotions as a discriminating factor for those with a high sense of life meaning. Low negative emotions correspond to low online shopping addiction (27.45%), while high negative emotions prompt further assessment based on a sense of place. A high sense of place in this context indicates online shopping addiction with considerable accuracy (85.71%), whereas a low sense of place suggests an absence of addiction (0%). Conversely, students exhibiting low social anxiety levels tend to demonstrate low online shopping addiction propensity (9.26%). This multi-tiered decision tree model elucidates the complex interplay of psychological and environmental factors contributing to online shopping addiction among college students, offering valuable insights for targeted intervention strategies.

**Figure 3:**
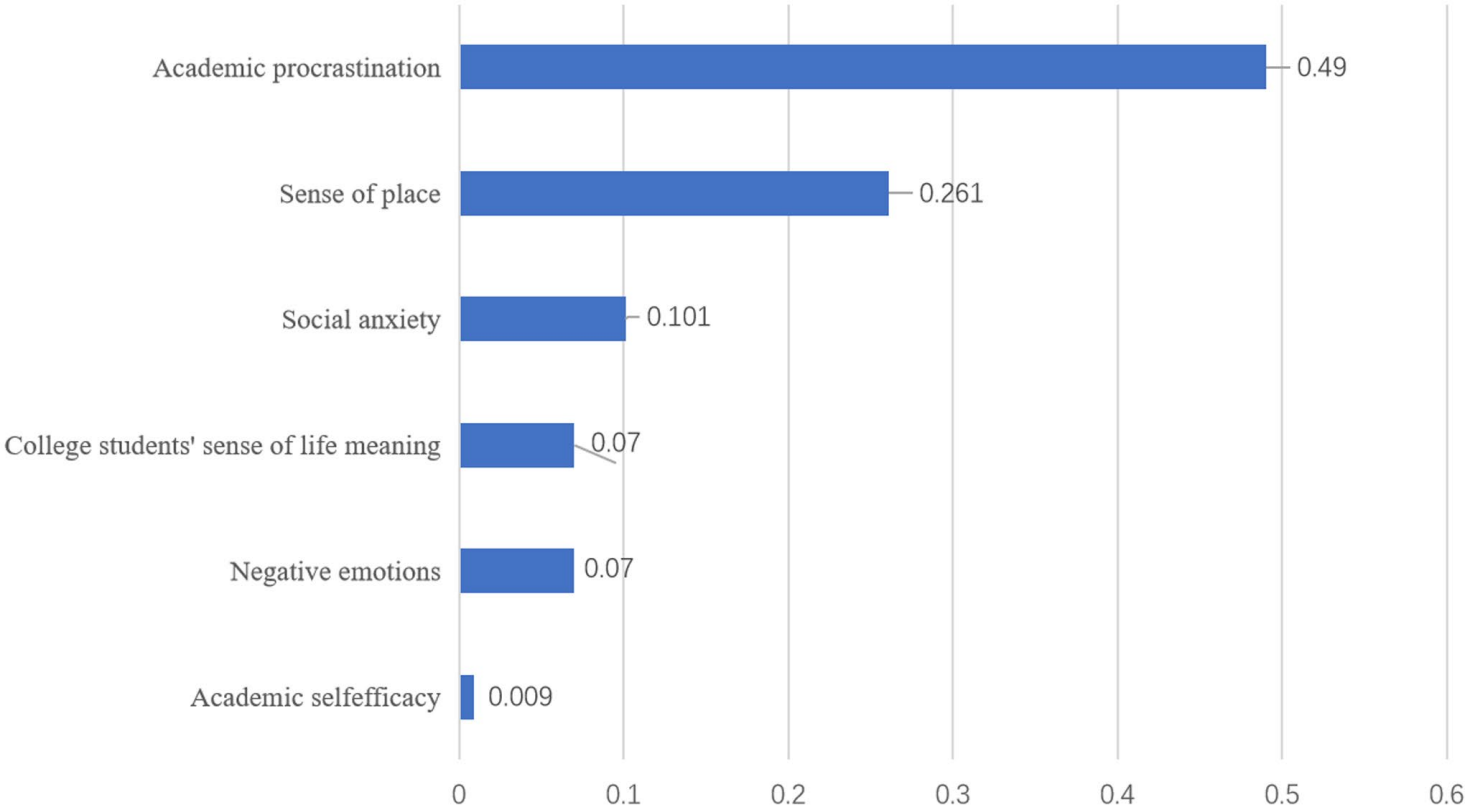
Predictor variables of online shopping addiction behaviors.

Figure 3 illustrates the relative importance of predictor variables in the model, quantifying their respective contributions to predicting online shopping addiction. Academic procrastination emerges as the most significant predictor, followed by orientation attachment and social anxiety in the second and third positions. Among college students, sense of meaning in life and negative emotions occupy the fourth and fifth ranks of importance. Notably, College Academic Self-Efficacy demonstrates comparatively lower predictive significance within this model. This hierarchy of predictor variables provides valuable insights into the multifaceted nature of online shopping addiction among college students, potentially informing targeted intervention strategies.

### 4.3 Evaluation of the model

Tables 4, 5 present the model’s confusion matrix and classification accuracy, respectively. The model demonstrates an accuracy of 79.45% on the test dataset. Further evaluation metrics reveal a precision of 71.88% and a recall of 52.27% on the test dataset, calculated according to standard definitions. Collectively, these three-evaluation metrics indicate robust performance of the predictive model, underscoring its utility in identifying online shopping addiction among college students.

**Table 4:** Confusion matrix.

		**Predicted class**	
		Class = Low	Class = High
Actual class of training data	Class = Low	211	11
	Class = High	39	50
Actual class of testing data	Class = Low	93	9
	Class = High	21	23

Accuracy refers to the proportion of correctly classified samples out of all samples. That is, Accuracy = (93 + 23)/(93 + 9 + 21 + 23). 79.45%. Precision measures how many of the predicted positive samples are truly positive. Precision = 23/(9 + 23). 71.88%. Recall represents how many of the actual positive samples were correctly predicted. Recall = 23/(21 + 23). 52.27%.

**Table 5:** Classification accuracy.

Title 1	Title 2	Number	Proportion
Training data	Correct	261	83.92%
	Wrong	50	16.08%
	Total	311	
Testing data	Correct	116	79.45%
	Wrong	30	20.55%
	Total	146	

## 5 Discussion

### 5.1 Discussion of results

This study extends beyond previous research by revealing the hierarchical relationship between multiple psychological predictors of online shopping addiction. While previous studies examined online shopping addiction through limited pathways–such as Wang Q. et al. (2022) and Wang X. et al. (2022) investigating its mediating role between academic procrastination and negative emotions, and Luo et al. (2018) focusing on the mediating effects of self-control and negative emotions on compulsive buying behavior–our findings demonstrate how academic procrastination interacts with sense of place and social anxiety to create distinct pathways to addiction. These insights enable development of more nuanced, personalized intervention strategies targeting specific predictor combinations.

Employing the C5.0 algorithm decision tree model, this study developed a six-factor predictive model for online shopping addiction behaviors and examined the contributions of these factors. The findings reveal: (1) Academic procrastination, sense of place, social anxiety, college students’ sense of meaning in life, negative emotions, and College Academic Self-Efficacy significantly contribute to predicting online shopping addiction. (2) The importance of these six factors follows the order: academic procrastination, sense of place, social anxiety, college students’ sense of meaning in life, negative emotions, and College Academic Self-Efficacy. (3) The evaluation results indicate that the predictive model demonstrates satisfactory predictive performance, with an accuracy of 79.45% and precision of 71.88%, which are considered good performance metrics in behavioral prediction models. Additionally, the model’s AUC value of 0.657 surpasses 0.5, demonstrating its superiority over random guessing (Fawcett, 2006).

This study identifies academic procrastination as the most crucial among the six variables in the decision tree model, aligning with findings from Tras and Gökçen (2020), Wang Q. et al. (2022), Ti et al. (2022), and Cerniglia (2019). These studies have established that academic procrastination positively predicts internet addiction. As a subtype of internet addiction, online shopping addiction’s predictive factors, including academic procrastination, corroborate previous research findings. One potential explanation for this phenomenon is that procrastinators may be less inclined to discontinue engaging in pleasurable activities, whether online, such as online shopping, or offline pursuits seeking gratification. Individuals with smartphone addiction may find it particularly challenging to cease device usage or manage interruptions (Zhang and Wu, 2020). Moreover, procrastination, including academic procrastination, is a personality trait that may constitute a risk factor for online shopping addiction. Procrastinators typically exhibit weak self-control and a preference for short-term rewards, rendering them more susceptible to internet addiction (Shi, 2018; Ti et al., 2022). Consequently, it is reasonable to consider academic procrastination a significant factor in predicting online shopping addiction. Recent research further supports our findings regarding the predictive power of academic procrastination and social anxiety. A longitudinal study by Hong et al. (2021) found that academic procrastination significantly predicts problematic mobile phone use among Chinese adolescents through distraction cognitions. Similarly, Rose and Dhandayudham (2014) revealed that social anxiety and negative emotional states were strongly associated with increased online shopping addiction tendencies, corroborating our model’s identification of social anxiety as a key predictor.

Furthermore, the sense of place emerges as the second most important predictor in the model. Chemnad et al. (2023) identified the school environment as a significant negative predictor of adolescent online shopping addiction. The school environment represents a relatively stable attribute shaping students’ behavior, contingent upon their perception of school conduct (Li et al., 2016). For college students, emotional attachment and degree of identification with the school can be considered manifestations of a sense of place. Typically, the absence of a sense of place may lead to dissatisfaction with real life and feelings of loneliness, prompting individuals to seek satisfaction and belonging in the virtual world. When college students experience loneliness, they become more susceptible to developing addiction to the internet’s virtual realm, indulging in gaming, or engaging in online shopping (Yu et al., 2019). A plausible explanation for this phenomenon is that the lack of real-world social interaction and sense of belonging may drive them to become addicted to virtual activities like online shopping to fill psychological voids and alleviate anxiety.

Additionally, social anxiety emerges as the third most important factor. Previous research has demonstrated that social anxiety significantly and positively impacts smartphone addiction (Xiao and Huang, 2022; Zhou et al., 2021). This is attributed to social anxiety making students reluctant to face real social situations. Online shopping offers a virtual environment where individuals can fulfill their shopping desires without encountering others. Those grappling with social anxiety often find it challenging to regulate their emotions through conventional social interactions and may struggle to alleviate anxiety and negative emotions effectively (Hou et al., 2021). Shopping can serve as a temporary outlet for relieving anxiety and facilitating emotional regulation, rendering online shopping an avenue for escaping social anxiety. Furthermore, individuals with social anxiety frequently experience diminished subjective well-being during social interactions, whereas online shopping provides a relatively secluded environment that enables them to evade social pressures and enhance their subjective well-being momentarily. This method of circumventing real social pressures may heighten individuals’ susceptibility to developing an addiction to online shopping to attain temporary psychological solace and gratification (Taþ and Güneþ, 2019), thereby escalating the risk of online shopping addiction.

Furthermore, the sense of life meaning among university students emerges as the fourth most important factor in this study. Research by Uzarska et al. (2021) suggests that shopping addiction is often driven by individual focus values such as self-enhancement and openness to change. To some extent, shopping addiction can be perceived as a pursuit in which individuals seek to attain an ideal lifestyle with minimal exertion while maintaining positive and healthy social connections. Particularly within the university student population, online shopping addiction, as a subtype of shopping addiction, may be driven by its ability to satisfy symbolic, experiential, and functional needs, thereby promoting self-identity through an ongoing process (Hou and Yang, 2021). Similarly, Dittmar et al. (2007) revealed that individuals who pursue emotional and identity enhancement through materialism are predisposed to display pronounced compulsive buying tendencies. Additionally, research by Wang X. et al. (2022) suggests that various hedonic motives, such as the pursuit of gratification and engaging in creative shopping, act as significant drivers for compulsive online shopping behavior. For instance, Yoo (2007) uncovered that Korean housewives perceive shopping as entertainment and a responsibility, viewing it as a way to enhance their quality of life. To and Sung (2014) proposed that online shoppers value privacy and a sense of achievement, with their greatest hedonic value being the ability to interact with others while shopping. Therefore, university students with a higher sense of life meaning may be more prone to developing online shopping addiction, reflecting a positive correlation between the sense of life meaning and online shopping addiction. This relationship highlights the role of personal values, efforts to maintain social relationships, and the pursuit of an ideal lifestyle in behavioral choices.

Moreover, negative emotions represent the fifth factor of relative importance. Research has found that negative emotions predict smartphone addiction (Gao et al., 2022; Meng et al., 2023). As a subtype of smartphone addiction, the predictive factors displayed in online shopping addiction also include negative emotions, corroborating previous research (Nawodya and Kumara, 2022; Rose and Dhandayudham, 2014; Wang Q. et al., 2022). Building on prior research, it can be inferred that under the influence of negative emotions, individuals may be more inclined to seek immediate psychological comfort through activities like online shopping, thereby exacerbating their dependence and addiction to online shopping. Additionally, negative emotions may impact individual executive functions, including decision-making, self-control, and impulse control. Anxiety, in comparison to neutral emotions, significantly impairs executive functions, which may consequently affect an individual’s ability to exercise self-control, potentially leading to addictive behaviors such as online shopping addiction (Shields et al., 2016).

Academic self-efficacy emerges as the sixth predictive factor. Saleh (2019) identified a positive correlation between students’ internet addiction and College Academic Self-Efficacy. Given that online shopping addiction falls under the umbrella of internet addiction, it is plausible that there exists a predictive relationship between online shopping addiction and College Academic Self-Efficacy. This is supported by Hassan et al. (2015), who found that College Academic Self-Efficacy positively predicts online shopping addiction. Delafrooz et al. (2011) observed a positive correlation between self-efficacy, trust, security, and online purchase intention. One possible explanation for this phenomenon is that self-efficacy positively influences trust in online vendors, subsequently affecting the willingness to engage in online transactions (Kim and Kim, 2005). Consequently, College Academic Self-Efficacy fosters trust in social media or shopping platforms, heightens the propensity to make online purchases and exerts a positive influence on online shopping addiction (Dash and Saji, 2008).

### 5.2 Significance

This study provides valuable insights into online shopping addiction among college students, holding both theoretical and practical significance. From a theoretical perspective, the decision tree model reveals predictive factors of online shopping addiction, thus offering avenues for a deeper understanding of addictive behaviors. This contributes to a more comprehensive understanding of the etiology and associations of various addictive behaviors within the field of psychology. These findings align with broader psychological theories of behavioral addiction, where Kuftyak (2022) suggests that academic procrastination serves as a significant stress factor impacting student behavior and academic performance. The prominent role of sense of place in our model supports Estévez et al. (2017) theory that attachment patterns significantly influence non-substance addictive behaviors. For university counseling services, this suggests the need for integrated intervention approaches combining time management training with environmental adaptation support. Furthermore, the model serves as a foundation for future research. Based on these identified factors, researchers can further explore the complex relationships between online shopping addiction and academic procrastination, sense of place, and social anxiety, potentially leading to more precise intervention measures in the field of mental health.

In practical terms, this study offers several significant applications. Firstly, by utilizing predictive factors such as academic procrastination, sense of place, and social anxiety, early detection of online shopping addiction risk becomes feasible. This enables targeted interventions, the promotion of healthy online habits, and the mitigation of addiction likelihood. Furthermore, educational institutions, mental health organizations, and parents can provide tailored support based on students’ addiction risk profiles. This may include time management and geographic adaptation training for students exhibiting high academic procrastination and low sense of place, as well as social skills training and psychological support for those with high social anxiety. Moreover, targeted strategies in public awareness campaigns and parental guidance can enhance community understanding of online shopping addiction issues and contribute to reducing its prevalence.

### 5.3 Limitations and future directions

This study acknowledges three primary limitations. First, its cross-sectional design only captures patterns of online shopping addiction prediction at a specific point in time. Second, the sample (*n* = 457) is drawn from a single university in China, potentially limiting the generalizability of the findings and necessitating further testing of the predictive model’s robustness in diverse regional contexts. Future research could address this by collecting data from multiple institutions using a longitudinal approach. Although our decision tree model demonstrates satisfactory predictive performance, its reliability requires further validation. The relatively small sample size (*n* = 457) may limit the model’s statistical power and stability across different subgroups. Future studies should employ k-fold cross-validation with larger samples and conduct sensitivity analyses to establish the model’s robustness. Additionally, comparing the decision tree’s performance with other machine learning approaches would help validate our methodological choice. Third, the study’s scope is constrained by the inclusion of only six factors influencing online shopping addiction, suggesting room for model refinement. Future investigations could enhance the predictive model by employing dynamic tracking methodologies, expanding sample size and diversity, and incorporating additional predictive factors to provide a more comprehensive understanding of online shopping addiction among college students.

The research findings propose a series of recommendations for preventing online shopping addiction among college students. Firstly, educational institutions and families are encouraged to monitor students’ academic procrastination tendencies, sense of place, and social anxiety levels. Secondly, for students exhibiting high levels of academic procrastination, it is advisable to cultivate practical time management skills. Thirdly, adaptive support should be extended to students with a low sense of place to enhance their connection to their academic environment. Fourthly, students experiencing high levels of social anxiety may benefit from targeted social skills training and psychological support interventions. Finally, institutions can implement personalized counseling programs, promote multicultural exchange opportunities, and enhance students’ overall life satisfaction to mitigate the risk of online shopping addiction.

## 6 Conclusion

This study established a six-factor predictive model for online shopping addiction using the decision tree algorithm. The results demonstrate that this model can effectively assess and predict students’ online shopping addiction behaviors, with an accuracy rate of 79.45%. The predictive model reveals three critical predictors of online shopping addiction: academic procrastination, sense of place, and social anxiety. Additionally, the sense of life meaning, negative emotions, and College Academic Self-Efficacy among college students also possess predictive value for online shopping addiction, albeit to a lesser extent. These findings contribute to a more nuanced understanding of the complex factors influencing online shopping addiction among college students and provide a foundation for developing targeted prevention and intervention strategies.

## Data Availability

The raw data supporting the conclusions of this article will be made available by the authors, without undue reservation.
